# 3D Printing of Organs-On-Chips

**DOI:** 10.3390/bioengineering4010010

**Published:** 2017-01-25

**Authors:** Hee-Gyeong Yi, Hyungseok Lee, Dong-Woo Cho

**Affiliations:** Department of Mechanical Engineering, Pohang University of Science and Technology (POSTECH), Pohang, Kyungbuk 37673, Korea; yhg0709@postech.ac.kr (H.-G.Y.); albert1222@postech.ac.kr (H.L.)

**Keywords:** 3D printing, cell-printing, bioprinting, organ-on-a-chip, in vitro tissue model, in vitro disease model

## Abstract

Organ-on-a-chip engineering aims to create artificial living organs that mimic the complex and physiological responses of real organs, in order to test drugs by precisely manipulating the cells and their microenvironments. To achieve this, the artificial organs should to be microfabricated with an extracellular matrix (ECM) and various types of cells, and should recapitulate morphogenesis, cell differentiation, and functions according to the native organ. A promising strategy is 3D printing, which precisely controls the spatial distribution and layer-by-layer assembly of cells, ECMs, and other biomaterials. Owing to this unique advantage, integration of 3D printing into organ-on-a-chip engineering can facilitate the creation of micro-organs with heterogeneity, a desired 3D cellular arrangement, tissue-specific functions, or even cyclic movement within a microfluidic device. Moreover, fully 3D-printed organs-on-chips more easily incorporate other mechanical and electrical components with the chips, and can be commercialized via automated massive production. Herein, we discuss the recent advances and the potential of 3D cell-printing technology in engineering organs-on-chips, and provides the future perspectives of this technology to establish the highly reliable and useful drug-screening platforms.

## 1. Introduction

A microenvironment provides a niche with crucial factors for cells to interact, grow, differentiate, and function. Tissue culture plastics such as dishes and flasks are very common and convenient to perform the expansion and maintenance of cells and high-throughput drug screening (e.g., 96- and 384-well plates). However, these flat and simple environments poorly reflect the key elements of an actual body, for example, 3D arrangement, softness, elasticity, mechanical stimuli, fluid flow, and extremely diverse communications (autocrine, paracrine, and endocrine signaling). However, by using in vitro culture platforms, we can precisely control the experimental conditions and utilize various assays for in-depth analysis. Therefore, 3D culture platforms, which can provide both biomimetic microenvironment and controllable experimental conditions, are necessary to understand the mechanisms of disease progression and to find an appropriate treatment strategy.

Organs-on-chips have come into the spotlight with their capability to replicate organ-level functions by introducing cells into a microfluidic device that includes precisely fabricated chambers and channels. The microfluidic device serves as a bioreactor that engineers the cells by reproducing the biomimetic stimuli, both dynamic mechanical cues (e.g., rigidity [1] and fluid flow [2]) and chemical cues (e.g., chemotaxis [3] and oxygen gradients [4]), to the microengineered tissues [5]. Organ-on-a-chip engineering focuses on reproducing the minimized essential functions of the target organ. Lung-on-a-chip [6] is a typical example of this concept. This chip reconstitutes the permeable and functional alveolar barriers experiencing a specific stimulation from the stretching motion due to breathing. Likewise, the field has recently shown eye-opening progress for modeling tissues such as intestinal tissue [7], blood–brain barrier [2], and bone marrow [8]. Now, organs-on-chips ultimately aim to establish a body-on-a-chip by interconnecting different organs such as the liver, bone marrow, and a tumor to produce an experimental set with a systemic interaction for screening drugs and generating replacements for diseased or damaged organs [9,10].

**Figure 1:**
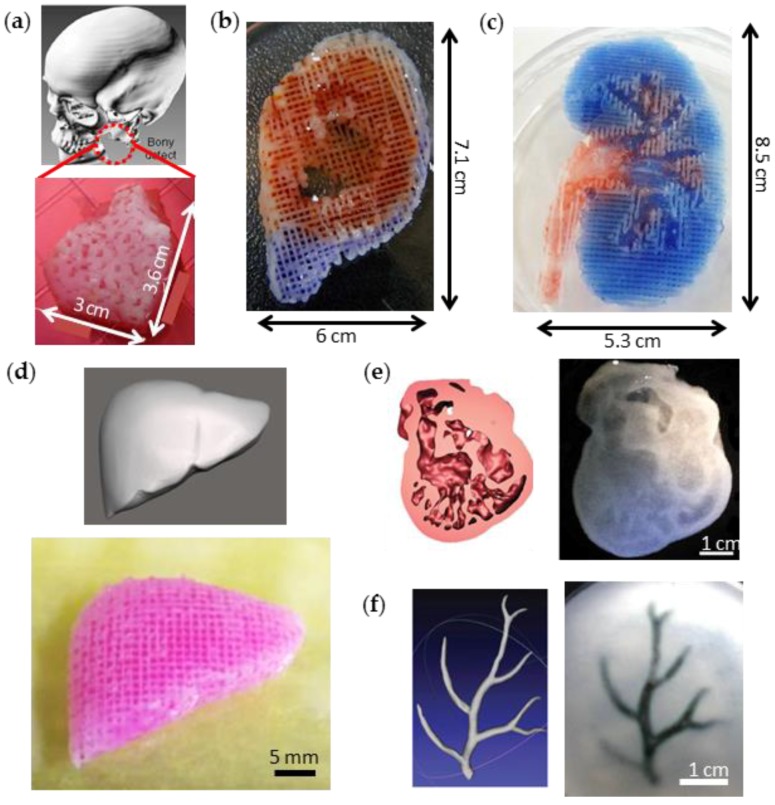
3D printing of biological constructs with heterogeneous and complex structures. Photographs of the 3D-printed (**a**) a mandible bone construct, (**b**) an ear cartilage with ear lobule, (**c**) a kidney with renal pelvis, (**d**) a liver, (**e**) a heart cross-section, and (**f**) an arterial tree. Reproduced with permissions from [18,19,21,22,24].

3D printing is an emerging field in diverse areas, including medicine [11], tissue engineering [12,13,14], electronics [15,16], and aerospace engineering [17]. This is because 3D printing not only enables the building of various and complex structures through a layer-by-layer process but also the adoption of various materials. This technology is a striking method in tissue engineering research that builds 3D scaffolds with patient-specific shape and complicated porous design [18] and to create living tissue constructs such as bone [19], ear cartilage [20], liver [21], and so on (Figure 1). The pre-fabrication of 3D scaffolds with printing accompanies wider options for selecting materials and follows a top–down approach, seeding the cells onto the scaffolds from the outside prior to implantation. The 3D printing of living tissue constructs follows a bottom–up approach to spatially manipulate the cells and to generate a heterogeneous structure with multi-material. The 3D cell-printing (also called 3D bioprinting) facilitates the construction of anatomically and physiologically relevant tissues by the precise patterning and layering of various cellular compositions and biomaterials [19,22,23].

Furthermore, 3D printing is gaining attention for the fabrication of microfluidic devices. This technology is capable of creating channels with complex designs and lure or barb connectors under a one-step fabrication process. Thus, it has emerged as a way to produce microfluidic devices with an automated and assembly-free 3D fabrication process [25,26]. Therefore, 3D printing of a microfluidic device, as well as the living constructs in it, can be a promising method to generate organs-on-chips in a simpler way, but with more sophisticated heterogeneous tissue. With the convergence between 3D printing and organs-on-chips engineering, we probably can create complex artificial tissues with the proper microarchitecture for mechanical and chemical stimuli, and thereby, construct an advanced platform performing human-like functions. By doing so, 3D printing technology promises to lead organ-on-a-chip engineering into the next generation.

In this paper, we discuss the possibilities of 3D printing for producing physiologically relevant organs-on-chips. We first introduce the current techniques (printing materials and working principles) of 3D cell-printing technologies for fabricating organs-on-chips. We then review the recent advances in printed organs-on-chips from the aspects of physiological relevance and manufacturing technology. Finally, to incorporate 3D printing technology into organ-on-a-chip engineering, we suggest several considerable strategies to improve 3D-printed organs-on-chips from various aspects of materials, printing techniques, instrumentation, personalization or customization, and commercialization in the future.

## 2. Current Techniques for 3D Cell-Printing

### 2.1. Printing Materials

In 3D printing of an organ-on-a-chip, the printing ink can be any biocompatible material, depending on the purposes and functions of the chip components. Printing inks can be broadly divided into two categories, natural and synthetic. The biological, chemical, and mechanical characteristics differ between the two categories. We describe the representative materials for printing organs-on-chips.

#### 2.1.1. Natural Materials

Natural materials originate from various living organisms and exhibit highly biocompatible characteristics. These materials—such as alginate, gellan gum, collagen, fibrin, and gelatin—usually form hydrogels, called bioinks, and are used to encapsulate cells in 3D cell printing. Bioinks have a viscoelastic property and high water content, and protect the cells during the printing process. The cells encapsulated in the hydrogels are insulated from exogenous risk factors such as mechanical stress when passing through the printing nozzle, drying, and potential contaminating factors from the printing space [27,28].

Natural materials from marine algae (e.g., alginate [29] and agarose [30]) and plants (e.g., gellan gum [31] and cellulose [32]) are gel-forming polysaccharides. Because these materials can be massively synthesized from the engineered bacteria, they are abundant and low-cost. Additionally, the materials have easily tunable characteristics, including gelation kinetics and rheological properties [29,33], compared to mammalian-derived materials, and many investigators have adopted these as bioinks. The viscous solutions composed of these materials can be polymerized chemically or physically. Alginate and cellulose can be chemically cross-linked by adding cations such as calcium chloride or other metal salt solutions. Agarose and gellan gum show thermos-reversible gelation kinetics. However, these materials inherently have no site that interacts with mammalian cell membrane proteins. Thus, there are many studies on the modification of materials, such as immobilization of arginylglycylaspartic acid, on the polysaccharide chain [34,35,36].

Natural materials from mammalian tissues show especially high bio-affinity and bio-activity because their extracellular matrix (ECM) molecules bind directly to the transmembrane receptors of mammalian cells. As the most abundant ECM component in the human body, collagen fibril is widely used in in vivo and in vitro experiments. Collagen monomers self-assemble into a fibrillar structure and entangle a viscoelastic gel as the temperature, pH, and ionic strength approach physiological conditions [37]. Moreover, the network structure and mechanical properties of collagen gel can be tuned by adding secondary gel components or cross-linkers (e.g., 1-ethyl-3-(3-dimethylaminopropyl)carbodiimide and n-hydroxysuccinimide [38]) [39]. On account of these features, collagen is an attractive bioink and suitable for various cell-printing and organ-on-a-chip applications [3,20].

Fibrin is a fibrous protein with a crucial role in blood clotting and hemostasis. It is generated by the action of thrombin on monomer fibrinogen. When thrombin releases fibrinopeptides from fibrinogen, the remnant fibrin monomers aggregate into insoluble fibrin. Because this reaction proceeds rapidly, fibrin is used extensively as a sealant in clinical treatments [40] and is useful in the cell-printing process. Although fibrin has an inherently low strength, its rapid gelation helps in maintaining the 3D shape of the printed cellular construct while the main material is fully polymerized. Using this mechanism, Hinton et al. successfully printed the entire brain- and heart-shaped structures by directly dispensing a collagen hydrogel containing both cells and fibrinogen into a gelatin slurry bath with thrombin [19].

Gelatin is mass produced by denaturation of collagen from animal skin and bone. Because gelatin is abundant, low-cost, and easy to handle, it is widely applied in in vitro experiments. The thermal cross-linking mechanism of gelatin is opposite to that of collagen. Gelatin normally dissolves at above 40 °C, and becomes gel below 30 °C due to random coil formation. Hence, gelatin cannot retain its shape at 37 °C, the temperature of typical in vitro culture environment. Therefore, synthesis of gelatin-methacrylate (GelMA) hydrogels has been studied to maintain the 3D morphology of the printed structure via UV-mediated polymerization even after increasing the temperature to 37 °C after printing under cool conditions [28].

With current advances in tissue engineering and artificial organ development, decellularized ECM (dECM) is recognized as an ideal material for reproducing the natural microenvironment of cells in native tissues [41]. Although single-component, purified natural materials can be combined in various ways, these combinations cannot fully replicate the heterogeneous and various configurations of ECM components in actual tissues. On the other hand, dECM preserves many components of individual tissues—such as proteins, proteoglycans, and cytokines—thus demonstrating excellent potential for inducing tissue-specific cell differentiation and growth. By exploiting these superior features of dECM, our group has pioneered the development of dECM bioinks [42,43,44]. The cross-linking mechanism of bioinks made of heart, cartilage, and adipose tissue dECMs is similar to that of collagen, with rheological characteristics that enable 3D printing. Moreover, in each of these bioinks, the stem cells differentiate into a tissue-specific lineage. Choi et al. demonstrated that muscle dECM bioink produces tunable and complex shapes, and generates more matured and functional muscles than single-collagen bioink [44].

#### 2.1.2. Synthetic Materials

Synthetic materials are tailorable for a particular purpose and are consistent from batch to batch. The biocompatible synthetic polymers exhibit low cytotoxicity and bioinert property. Since most of these materials show higher stiffness and rigidity than natural hydrogels, they are able to serve as a cell-supporting framework for 3D cell-printing. In addition, the biocompatible polymers with non-degradable properties are a promising materials for constructing the housing parts of entire organs-on-chips. We introduce some of the representative synthetic polymers capable of printing organs-on-chips.

Polycaprolactone (PCL) is an FDA-approved thermoplastic polymer that is widely used in sutures, implantable devices, and other biomedical applications [45]. Although this polymer has biodegradable characteristics, the total degradation period exceeds one year, and it maintains its shape over the usual test period of in vitro experiments [46]. PCL has an advantage of a relatively low melting temperature (above 60 °C) and is suitable for extrusion printing of the framework part directly interfaced to the cell-printed material. When the molten PCL is extruded from the printing nozzle, the temperature decreases to slightly above the body temperature and the PCL solidifies rapidly. Using this phenomenon, our group has proposed a printing method that reinforces the 3D cell-printed construct by alternately printing PCL frameworks and cell-containing bioinks [20,22,47]. With our own developed multi-head deposition system and multi-tissue/organ building system, we also demonstrate that the printed PCL framework does not harm the printed cells in the bioink [46].

Silicone is non-degradable, remarkably flexible, and easily generated by mixing a curing agent with an elastomer base. It is extensively used for biomedical instruments (e.g., tubes, catheters, and gaskets) and implants (breast implants and drains). Soon after, Whitesides et al. proposed the soft lithography method [48], PDMS became popular in generating microfluidic devices and cell-culturing devices [5,26]. The unique flexibility and toughness allows PDMS to be removed from precisely fabricated wafers with microscale features. In addition, the transparency of this material is useful to visualize the cultivated tissue or transport targeted particles. However, there have been major challenges involved in the commercialization of PDMS chips because PDMS molding has been largely a labor-intensive process. To overcome this limitation, printing of PDMS was attempted by extrusion printing. Because silicones can reversibly or irreversibly bond to glass, plastic, and other materials, they could be printed as an outer wall on a specific substrate, providing storage for hydrogels or culture media. Lewis’ group cultivated vascular networks [49] and a kidney proximal tubule [50] within the defined chambers of silicone printed on glass.

Pluronic F127 is a triblock copolymer consisting of two hydrophilic poly(ethylene oxide) (PEO) blocks and a hydrophobic poly(propylene oxide) (PPO) block, which is arranged in a PEO-PPO-PEO configuration. Above the critical temperature (~4 °C), Pluronic F127 forms micelles in water and exhibits a gel-like viscoelastic property. Conversely, below the critical temperature, the micelles become soluble in water and are rapidly liquefied. This characteristic of Pluronic F127 has been utilized in perfusable channels surrounded by very soft hydrogel. Kolesky et al. printed a highly tortuous vascular-like channel from this material [49]. Gel-like Pluronic F127 was printed as a sacrificial part on a pre-casted hydrogel base. It was embedded within the hydrogel to cover the printed channel and was finally flushed out by cold media after irreversible gelation of the hydrogel. By the same methodology, Homan and Kolesky et al. fabricated a perfusable proximal tubule [50].

There are also several synthetic materials available to print the housing part and mechanical elements. Photo-curable resins such as Watershed [25], Visijet SL Clear [51,52], PEG-DA [53], and MED610 [26] can be incorporated in 3D printing systems with laser- or visible light-mediated polymerization. They are less flexible and less gas-permeable than PDMS, but remain transparent to obtain optical clarity. Thermoplastic polymers such as acrylonitrile butadiene styrene [54] and cyclic olefin copolymer [55] are adaptable to extrusion-based printing and provide clarity.

### 2.2. 3D Cell-Printing Methods

3D printing technology has been used in many areas including industry and research since the 1980s. Many manufacturing and molding methods have been replaced by 3D printing technology and there have been several developments in this field [56,57]. With recent advancements in precise cell/ECM positioning, 3D printing has emerged as an effective technology for preparing complex biological structures. 3D printing methods include micro-extrusion, inkjet, and laser-assisted printing, each of which is briefly discussed below.

#### 2.2.1. Micro-Extrusion Printing

**Figure 2:**
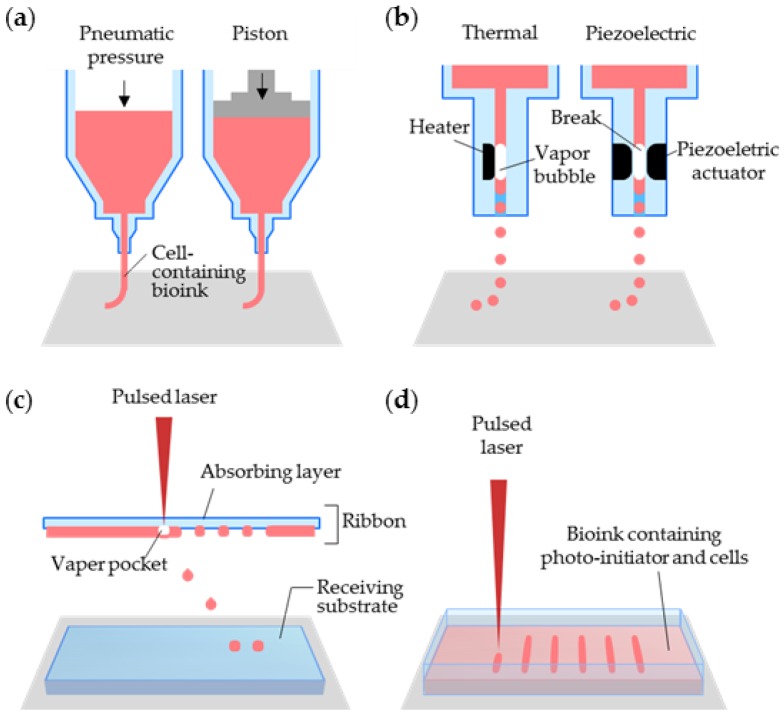
Schematics of 3D-cell printing methods with different working principles: (**a**) micro-extrusion, (**b**) inkjet, (**c**) laser-assisted printing, and (**d**) stereolithographic printing. Reproduced with permission from [76].

Biological 3D structures are most commonly printed by micro-extrusion, which directly deposits the printed materials onto a substrate by using a micro-extrusion head (Figure 2a). Under physical forces, the biomaterials and cells can be selectively dispensed at their intended positions through nozzles and needles. The force can be applied through pneumatic pressure [21,58] or a mechanical load that is exerted by a piston [20,59,60]. The micro-extrusion-based system is equipped with multiple printing heads containing different cells/bioinks for preparing complex heterogeneous structures [22,61]. When using the multiple heads, we should consider the position and spacing of the nozzles, the printing speed, the dispensing forces, and the nozzle diameters. The bioinks must also be sufficiently viscous to maintain the 3D shape of the construct. Although micro-extrusion can deposit bioinks over a wide range of viscosities, a high-viscosity bioink prevents the collapse of the printed construct and enables high-resolution printing.

#### 2.2.2. Inkjet Printing

The inkjet printing method delivers a controlled volume (droplets) of cell-suspended liquid at a pre-defined position. The liquid is vaporized into microbubbles by an electrically heated nozzle [62] or a piezoelectric actuator [63,64], and then exits the nozzle as droplets (Figure 2b). Electrically-heated inkjet printing delivers high printing speed at a low cost, but exposes the cells to heat and cannot properly control the droplet size [12]. Although inkjet printing with a piezoelectric actuator can resolve these problems, the actuator frequencies (15–25 kHz) can damage the cell membrane and lyse several sensitive primary cells [65]. Without these, there are multiple reports that show excellent cell viability after the inkjet printing process [66,67,68,69]. Last, a wide range of viscous materials can be used in inkjet printing. However, the inkjet printing method is best suited for the low-viscosity range (~0.1 Pa·s) of bioinks [70]. Overall, inkjet printing improves the resolution of the cell droplets over the micro-extrusion printing method, but cannot print large-scale biological structures. Despite its disadvantages, inkjet printing is favored for replicating narrow complex biological structures because it offers high-resolution droplet printing.

#### 2.2.3. Laser-Assisted Printing

In laser-assisted printing systems, biological structures are patterned or prepared by laser-induced forward transfer [71] (Figure 2c). Laser-assisted printing overcomes some of the limitations of micro-extrusion and inkjet printing [72]. For example, it offers the highest resolution of droplets due to the accuracy of laser targeting itself. In the first step of laser-assisted printing, the laser is focused onto a laser-absorbing support layer, called the ribbon. In the second step, the cell-laden hydrogel beneath the ribbon is bombarded with laser pulses. Finally, the liberated cell droplets are printed on the receiving substrate [73,74]. The resolution of laser-assisted printing is affected by many factors such as laser power, thickness of the biological layer, and the gap between the ribbon and the receiving substrate. Even though laser-assisted printing shows the highest resolution, many factors still need to be adjusted.

Another type of laser-assisted printing is stereolithography (SLA), which is the oldest and one of the most powerful 3D printing techniques capable of producing complex 3D structures. The basic mechanism of SLA is solidification of the liquid photopolymer by laser-induced photopolymerization (Figure 2d), typically at ultraviolet, infrared, or visible wavelengths [75]. After the photopolymerization of 3D patterns of 3D models, 3D structures can be obtained by a layer-by-layer process [75]. The laser pulse solidifies the material (the combined bioink, cells, and photo-initiator) at the reservoir, and finally, stacks the 3D-patterned solidified layers into a 3D biological construct.

## 3. Applications of 3D Cell-Printing to Tissue Models

### 3.1. 3D Cell-Printined Organs-On-Chips with Static Culture

3D cell printing is a technology that facilitates the construction of complex 3D histological structures and generates functional living tissues and artificial organs [77]. While still in its beginning stages, 3D cell printing has demonstrated its potential use in testing or screening of drugs by modeling tissues and diseases, including skin [78], liver [79,80,81], and cancers [82,83] (Table 1). These organs-on-chips are printed to generate the biomimetic heterogeneous structure and are cultivated under static conditions to maturate the tissues. Although mechanical dynamic stimuli are excluded in those organs-on-chips, the heterogeneous constructions have induced feasible responses against the tested drugs.

Skin is a multilayered barrier that covers the whole body. It plays various important roles such as protecting internal organs from external threats, maintaining homeostasis by thermoregulation, removing waste products, and sensing external stimuli. The multiple layers of skin contain various elements performing diverse functions (e.g., papillae, sweat glands, hair follicles, and pigments). Hence, a functional in vitro skin model must recreate the distinct multilayered structure of skin. Although the dermis and epidermis layers may be generated by manual sequential seeding of fibroblasts and by co-culturing keratinocytes in an air–liquid interface (ALI) environment, respectively, this approach is limited by nonuniform thickness and intermittent reproduction. In contrast, the 3D printing approach can create multilayer structures with the desired thicknesses and patterns. Applying an in-house microvalve-mediated droplet printing system, Lee at al. printed a human skin tissue model by fabricating two layers of keratinocytes, followed by a repeatable stacking of double layers of a dermal matrix layer and a fibroblast layer (total > 10) [78]. After one to two weeks in an ALI culture, the skin maturation was observed by epithelization and stratification. More importantly, Lee et al. demonstrated that 3D printing avoids significant shrinkage and concavity development in the skin model, which demerits the manual seeding method.

**Figure 3:**
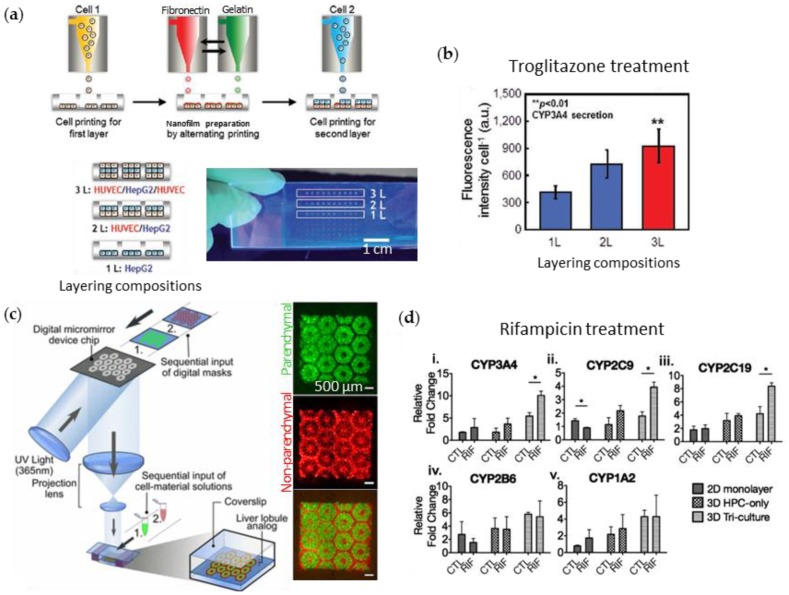
3D cell-printed livers-on-chips. (**a**) Schematic diagram of inkjet printing into multiple micro-wells to create liver tissues with three layering compositions: 1L (HepG2), 2L (HUVEC/HepG2), and 3L (HUVEC/HepG2, HUVEC); (**b**) Hepatotoxic responses with increase in CYP450 3A4 (CYP3A4) secretions under troglitazone treatment; (**c**) Illustration of the sequential SLA process for building the liver lobule structure and (**d**) rifampicin-induced changes in expression of the CYP450 series in HPCs grown in the 2D monolayer, the 3D HPC-only model, and the 3D-printed model (3D Tri-culture). Reproduced with permissions from [79,80]

Liver is a vital metabolic organ responsible for detoxification, digestive biochemical production, and glycogen storage regulation. It is organized by parenchymal hepatocytes and various non-parenchymal supporting cells (such as Kupffer cells, Ito stellate cells, and endothelial cells). Liver functionality relies on the cell–cell communication between the parenchymal and non-parenchymal liver cells. To investigate this feature, Matsusaki et al. generates a chip containing multiple arrays of mini-human liver tissue by inkjet printing (Figure 3a) [79]. They stacked, with the fibronectin-gelatin film as a glue, the layers of hepatocytes (Hep G2) and human umbilical vein endothelial cells (HUVECs) with different layering compositions (a single layer of Hep G2 and double and triple layers of Hep G2 and HUVEC) and compared them. The metabolic function and detoxification activity was most elevated in the triple-layered tissue, where the hepatocyte layer is sandwiched between an upper and lower endothelial cell layer. When treated with the hepatotoxic drug troglitazone (Rezulin), the triple-layered model exhibited the highest cytochrome P450 (CYP450)-mediated metabolism among the three models (Figure 3b). Ma et al. attempted 3D printing of anatomical features as well as various cell configurations in the liver [80]. They fabricated the liver unit, a lobule structure, by projection-based 3D printing. They chose two bioinks: the 5% GelMA (~5 kPa compressive stiffness similar to healthy liver tissue) for the parenchymal tissue formation and the 25% GelMA/1% glycidal methacrylate-hyaluronic acid (GMHA; ~4 kPa compressive stiffness) for vascularization. The sequential projection technique generated a hexagonal liver lobule with a complex pattern that is composed of two parts, the parenchymal tissue part of the human-induced pluripotent stem cell-derived hepatic progenitor cells (hiPSCs-HPCs) and the non-parenchymal tissue part with radial structure of the supporting cells (HUVECs and adipose-derived stem cells) (Figure 3c). When it is treated with rifampicin, a potentially hepatotoxic antibiotic drug, the hexagonal-patterned tri-culture model synthesizes more CYP450 than the 2D monolayer and HPC-only models (Figure 3d). Nguyen et al. also tried to generate compartmented regions of parenchymal tissue and non-parenchymal tissue by extrusion printing [81]. They dispensed the NovoGel-containing hepatic stellates and HUVEC in the border line in the well, and then filled it with aggregates of hepatocyte by printing. They observed the visible compartmentalization and selective sensitive response to the hepatotoxic drug trovafloxacin and the non-toxic drug levofloxacin.

**Figure 4:**
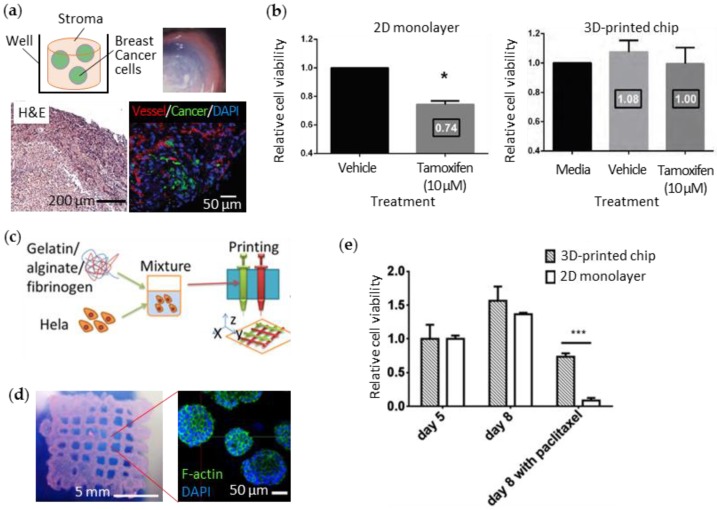
3D cell-printed cancers-on-chips (**a**) Schematic diagram and microscopic observations (lower left: H&E staining, lower right: immunofluorescent staining) of the extruded breast stromal cells and cancer cells into a multi-well plate; (**b**) Increased chemosensitivity to tamoxifen from breast cancer cells cultured in 3D-printed chip compared to those in a 2D monolayer; (**c**) Process of the micro-extrusion of cervical cancer cells (Hela) with gelatin-alginate-fibrinogen bioink; (**d**) Photograph of the printed cervical cancer-on-a-chip showing the lattice pattern and its fluorescent microscopic image showing the cell morphology; (**e**) Increased chemosensitivity to paclitaxel from Hela cells cultured in 3D-printed chip compared to those in a 2D monolayer. Reproduced with permission from [82,83].

Cancer is the most prevalent disease and remains difficult to treat. To reveal the exact mechanisms of drug action, in vitro cancer models ought to reproduce the extremely complex and heterogeneous characteristics of tumors. Interactions among the cancer cells, ECM, and the peripheral cells strongly affect the pathological progression and aggressiveness of cancer. King et al. generated artificial human breast cancer by extrusion printing to simulate the progression of the cancer in the breast stromal tissue [82]. They first printed the stromal clumps containing adipocytes, mammary fibroblasts, and endothelial cells into multiple wells in a culture plate. Next, they dispensed aggregates of breast cancer cells into the stroma by micro-extrusion printing (Figure 4a). The breast cancer cells surrounded by stromal tissue showed angiogenesis resulting from communication between the cells. When the common breast-cancer drug tamoxifen was applied to the 3D-printed breast cancer, the cells showed higher chemoresistance than those in the monolayer culture (Figure 4b). Zhao et al. printed an artificial human cervical cancer with porous 3D architecture to ensure the oxygen supply [83]. They extruded the fibrinogen-gelatin-alginate bioink (~11 Pa·s viscosity at 10 °C) containing human cervical cancer cells (Hela) in a lattice pattern (Figure 4c) and observed the realistic morphological changes (Figure 4d). The printed cervical cancer also showed lower sensitivity to paclitaxel (the typical anticancer drug) than 2D-cultured cancer cells (Figure 4e).

### 3.2. 3D-Printed Organs-On-Chips with Microfluidic Device

With growing interest in 3D printing of a microfluidic device, the printing of organs-on-chips with microfluidic channels is just steps away. There are two main approaches that integrate 3D cell-printed tissue and the microfluidic device: two-step fabrication and one-step fabrication. Two-step fabrication is the printing of micro-organs on the pre-fabricated microfluidic platforms. One-step fabrication is the printing of the entire chip device, including the cells and mechanical elements (e.g., a gasket and microfluidic channels), within a single process. We discuss in the following sections the 3D-printed organs-on-chips generated by these two strategies (Table 2).

**Figure 5:**
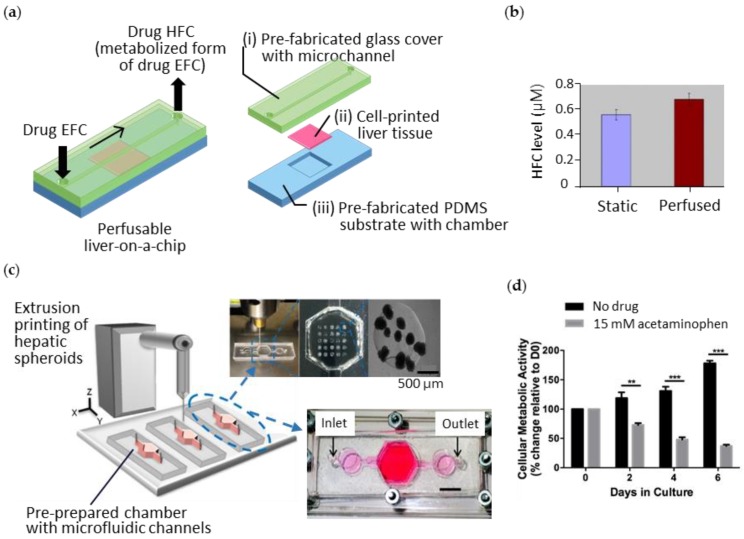
3D cell-printed liver constructs integrated with a pre-prepared microfluidic device. (**a**) Illustration of the 3D-printed perfusable liver-on-a-chip and its exploded view; (**b**) The higher level of metabolized drug 7-hydroxy-4-trifluoromethyl coumarin (HFC) under perfusion condition compared to that under static condition; (**c**) Schematics of extrusion printing of liver cells onto pre-prepared microfluidic device and its photographs, showing the array of hepatic spheroids in the chamber; (**d**) Hepatotoxic effect of acute acetaminophen on the liver tissue. Reproduced with permissions from [84,85].

The direct printing of living constructs on a pre-fabricated chip can facilitate the creation of heterogeneous designs of cellular constructs according to a specific purpose. This strategy allows the use of conventionally fabricated microfluidic chambers and channels with high resolution. Chang et al. generated a perfusable liver-on-a-chip to investigate drug metabolism (Figure 5a) [84]. By extrusion printing, they deposited the alginate bioink containing Hep G2 cells onto the pre-fabricated chamber in a PDMS substrate, and then assembled it with the glass cover containing microfluidic channels. They demonstrated that printing on the prepared microfluidic device enables structural adaptability of the cell constructs to the design specifications. The perfusable liver-on-a-chip was operated to metabolize the drug 7-ethoxy-4-trifluoromethyl coumarin into 7-hydroxy-4-trifluoromethyl coumarin. When they compared the drug metabolism rates of their artificial liver tissue with static and perfused conditions, the higher efficiency of the perfusion culture was confirmed (Figure 5b).

Bhise et al. also printed the perfusable chip device containing the multiple micro-liver tissues within it using extrusion printing (Figure 5c) [85]. They deposited GelMA bioink containing hepatic spheroids onto the casted PDMS chambers and assembled with the pre-fabricated microfluidic channels [85]. They utilized the hepatic spheroids to enhance the self-secretion of ECM components and conducted perfusion cultures to maintain the viability of the cells inside the spheroids. In this chip, the hepatocytes secreted liver-specific markers and showed decreased metabolic activity resulting from acetaminophen treatment (Figure 5d). Likewise, the two-step approach for integrating microfluidic platforms with the 3D cell-printed tissues affirmed the results for future organ-on-a-chip development. However, it still required manual intervention, causing difficulties in automation, inaccuracy in reproduction, and contamination in cultivation, which might create hurdles when trying to commercialize the chip device.

**Figure 6:**
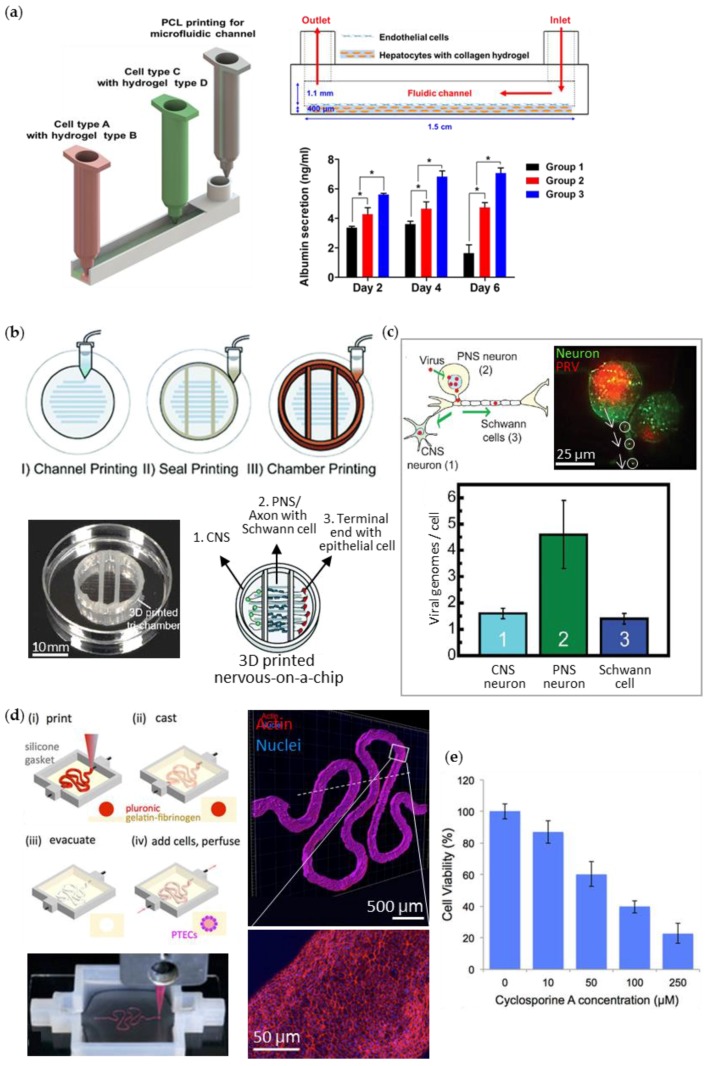
3D-printed organs-on-chips in a one-step fabrication process. (**a**) Schematic diagrams for extrusion printing of liver-on-a-chip resembling sinusoid (Group 1 = 3D cell-printed hepatocytes alone in static culture, Group 2 = 3D cell-printed hepatocytes–endothelial cells in static culture, and Group 3 = 3D cell-printed liver-on-a-chip); (**b**) Extrusion-printed nervous system-on-a-chip with compartmentalized chambers: central neurons (CNS) in chamber 1, peripheral neurons (PNS), and the axons with Schwann cells in chamber 2, and terminal cell junctions with epithelial cells in chamber 3; (**c**) Study of viral infection in the neuron system. Microscopic observation of the pseudorabies virus (PRV) transmission and the level of the transported virus in each chamber; (**d**) Illustration (upper left) and photograph (lower left) of the extrusion-printing process of a kidney proximal tubule-on-a-chip and the immunofluorescent stained images of the tubule (upper and lower right); (**e**) Nephrotoxicity effect of cyclosporine A on kidney epithelial cells in the chip. Reproduced with permissions from [50,87,88].

On the other hand, the one-step fabrication approach to construct the entire organ-on-a-chip was demonstrated as an effective manufacturing methodology with high productivity [86]. This method enabled the cellular compositions and channel structure to be built in various designs to achieve the heterogeneity and complexity. Our group successfully accomplished the one-step fabrication of a spatially heterogeneous liver-on-a-chip using extrusion-based multi-material printing by the in-house printing system with multiple heads (Figure 6a) [87]. The entire chip device was generated by alternatively dispensing the biocompatible and hydrophobic polymer and the two bioinks containing cells. PCL was used for the housing and microfluidic channels. For the printing of the cells, the Hep G2-laden collagen was used for the 3D cellular construct, while the HUVEC-laden gelatin was used for 2D cell-monolayer formation after the removal of liquefied gelatin at the physiological temperature. The dispensing sequences and the path were programmed following the desired final design. We demonstrated the various designs of the printed liver tissues-on-chips and promising liver functionality.

Similarly, Johnson et al. displayed the potential use of extrusion-based 3D cell printing in modeling a nervous system with customization. They dispensed PCL onto the culture dish to create microchannels and deposited grease and silicone across the channels to build compartmentalized chambers (Figure 6b). Afterward, they dispensed four cell suspensions into each chamber: (i) rat embryonic hippocampal neurons into the first chamber for the central neuron system (CNS) development, (ii) rat embryonic sensory neurons and Schwann cells into the second chamber for the peripheral neuron system (PNS) and axon development, and (iii) porcine kidney epithelial cells into the third chamber for the formation of axonal terminal junction. The printed cells penetrated the grease part in the bottom-most layer, went through the PCL microchannels, and formed an interconnected nervous system. Using this chip, they investigated the preferential transmission of the pseudorabies virus (PRV) from the inoculated site of the cell body of PNS to the other cell body of CNS, or to the terminal end (Figure 6c).

Extrusion printing was also applied to generate the chip device, including a perfusable and permeable channel composed of living cells, mimicking the human kidney proximal tubule. Homan et al. achieved the printing of a kidney proximal tubule-on-a-chip by adapting a fugitive ink (Figure 6d) [50]. First, they printed the silicon gasket onto a glass substrate, and then dispensed the convoluted channel with Pluronic F127. Using the extrusion nozzle, the chamber was filled, with a fibrinogen-gelatin-CaCl_2_-transglutaminase bioink (~3.5 kPa elastic modulus similar to a healthy kidney cortex) containing fibroblasts to mimic the kidney ECM. After gelation of the ECM-like tissue, they perfused the culture medium to wash out Pluronic F127 and to generate the hollow channel. Finally, the epithelial cells (RPTEC/TERT1) for the proximal tubule were seeded inside the channel and showed in vivo-like morphology formation and improved albumin uptake ability. The printed kidney-on-a-chip also exhibited nephrotoxicity against cyclosporine A, an immunosuppressive drug, in a dose-dependent manner. As confirmed in these examples, 3D printing technologies would maximize the effectiveness of the manufacturing process when building whole organs-on-chips, while reducing human labor costs due to complex structure preparation.

## 4. Conclusion and Future Perspectives

3D printing allows a bottom–up approach and is very effective at fabricating micro-organs of heterogeneous and complex structures on a chip or entire organs-on-chips composed of various materials. To produce the micro-organs or the organs-on-chips, various materials and printing methods are considered. At this point, 3D-printed artificial tissues and organs-on-chips have demonstrated the ability of this technique to achieve the physiological relevance and can be applied to drug screening. However, this technology is still in its beginning stage and there are many aspects that must be addressed in the future.

To develop the interface between various tissues, different tissue-specific bioinks can also be applied in 3D-printed organs-on-chips. The interfaces, such as the neuromuscular junction and neurovascular unit, do not only have the heterogeneous cell types, functions, and structure but also dynamic interaction and compatibility between the different tissues. Thus, fabrication of the heterogeneous structure and maturation of various cells can be achieved simultaneously. Shim et al. demonstrated this concept as a promising method to generate osteochondral tissue [89]. They fabricated a cartilage part right above the bone part using the in-house extrusion-based multi-head printing system, with different bioink compositions but the same human mesenchymal stem cells. For the superficial cartilage formation, they used a collagen mix containing bone morphogenic proteins and the cells. For the subchondral bone tissue, they used the supramolecular hyaluronic acid containing transforming growth factor-β and the same cells. During the culture period of two weeks, they observed differentiation of the stem cells into chondrogenic and osteogenic lineages from each part. Likewise, it is possible to differentially maturate the cells by using the different dECM bioinks in each part to induce natural cell–ECM interactions resulting in tissue-specific differentiation.

To measure outputs, a few studies incorporated electronic elements into the organs-on-chips. Very recently, Lind and Busbee et al. attempted to print a readable heart-on-a-chip outfitted with electric sensors and circuits for measuring the contractile stress from the laminar cardiac tissue [90]. They printed the silicone gasket, the flexible cantilever sensors, and the circuits on a glass substrate, and then cultured neonatal rat ventricular myocytes after seeding the cells on it. The maturated laminar cardiac tissue displayed natural cyclic contraction, i.e., a beating motion, and caused deformation of the strain gauge of the flexible sensor. The deformation was transformed into electrical signals and processed to estimate the contractile stress in real time. When varying the spacing between the grooves on the substrate, the engineered cardiac tissue showed different contractile stress. Although this study accomplished the proof-of-concept for printing an electronic component into the cell culturing device, it was still challenging to find conductive materials that had biocompatible properties. They had to perform the additional evaporation process to remove the solvent formulated in the inks used in printing electronic components. The ink was prepared by mixing carbon black (for the high-resistance cantilever sensor) into a solution of urethane dissolved in a solvent and the other ink was formulated with silver flakes (for circuit) mixed with a solution of polyamide dissolved in a solvent. Therefore, the methodology for preparing printable conductive inks should advance to be solvent-free, biocompatible, and thereby, applicable to in situ printing with cell-laden bioinks.

Furthermore, the technical advances in 3D printing should produce organs-on-chips with high resolution, high accuracy, a high-throughput assay, and more varied laboratorial functions. SLA displayed promise for both 3D cell printing and fabrication of microfluidic devices. This technique allowed fabrication of channels with high resolution (~100 μm) [26] and a device with a smooth surface, resulting in high clarity. However, it was less applicable to multi-material printing for the full construction of organs-on-chips. Inkjet-printing methods demonstrated high-resolution printing of the cells in a droplet (~20 μm) [76]. However, it was not easily applied when building 3D structures by stacking the printed low-viscosity materials. Extrusion-based printing was advantageous to both multi-material fabrication and 3D complex structure construction, but it was a challenge to increase the resolution (current: ~350 μm channel) using this technique [26]. Instead, it may be possible to combine these printing methods and other techniques to supplement the limitation of each method and maximize the advantages. The other considerable techniques are fabrication of modular microfluidics, 3D microfluidic networks produced by molding from a 3D-printed structure, 4D printing, and decellularization of an entire organ. Lee et al. fabricated the LEGO-inspired microfluidic blocks by SLA and demonstrated their assembly through various ways without leakage [91]. The casted microfluidic channels from the 3D-printed architecture were demonstrated by fabricating a device with a helical coil channel and the other channel passing through the inside of the helical coil [92]. 4D printing is a technology that confers a printed construct the ability to change its shape or function with time under external stimuli on the printed 3D structure. Gladman et al. strikingly demonstrated the shape changes of a 3D-printed orchid-mimetic hydrogel construct by the reinforcement of the hydrogel with nanocellulose [93]. The 2D flat architecture was deformed to a ruffled structure and a helicoidal structure. Decellularization of a whole organ is a method to preserve the original structure and vasculature of the organ by continuous perfusion of the cell removal agents through the existing blood vessel [94]. The acellular tissues’ built-in 3D-printed microfluidics might be favorable for vascularization. These techniques can be adapted to the construction of more sophisticated organs-on-chips, including interconnected multiple organs-on-a-chip (body-on-a-chip), and eventually, organs-on-a-chip integrated with a lab-on-a-chip to perform in situ biochemical assays in a drug screening process.

Finally, 3D printing can create personalized in vitro models constructed from patient-derived cells or induced pluripotent stem cells (iPSCs). If we can combine complex structures exhibiting organ-level responses with the patient’s genetic information, we may realize a highly reliable patient-specific disease model. Such models can reveal novel treatment methods for refractory genetic and familial disorders. Moretti et al. developed patient-specific iPSCs for modeling long-QT syndrome causing sudden cardiac death [95]. Similarly, Carvajal-Vergara et al. generated iPSCs from patients with LEOPARD syndrome and characterized the patient-specific disease progression using the cells [96]. Availability of iPSCs has increased and the cells can be very useful to understand the patient-specific disease and find a cure. However, the development of a platform capable of supporting iPSC growth and maturation is at an early stage [97], and it is anticipated that organs-on-chips will play a leading role in the future.

Currently, there are several commercialized services for modeling of tissues and cancers with 3D printing from start-up companies. Organovo® provides drug-testing services using their self-developed 3D cell-printed mini liver tissues [98]. RegenHU manages BioFactory® to open the 3D printing of the skeletal muscle tissues to users [99]. N3D Biosciences, Inc., sells the 3D-printed and magnetically-levitated cancer spheroids in multi-well plates [100]. Advanced Solutions Life Sciences provides matching services with their collaborators for 3D tissue design and construction [101]. L’Oreal, a cosmetics company, has also found collaboration partners to develop the artificial skin tissue to replace animal experiments [102,103]. Most of the companies supporting the printing of customized microfluidic devices are based on SLA technologies. Nanoscribe [104], Bio3D Technologies [105], LightFab [106], and FEMTOprint [107] offer custom-made devices using proprietary or open-source materials with transparency. MiiCraft Printer based on digital laser processing is on sale for microfluidics from Creative CADworks [108]. To the best of our knowledge, only Dolomite sells the fused deposition modeling printer using a cyclic olefin copolymer, named Fluidic Factory 3D Printer [109]. Consequently, there are many growing companies that are based on 3D printing of artificial tissues or chip devices, but the 3D-printed organ-on-a-chip is yet to be commercialized. We expect that advances in technologies for printing more physiologically relevant organs-on-chips with highly upgraded functions will accelerate the commercialization of the tissue/organ models and the practical use of these products in drug development to overcome several refractory diseases of mankind.

## Figures and Tables

**Table 1:** Summary of 3D cell-printed organs-on-chips without microfluidics.

Target Model	Reference	Cell Types	Bioinks	Printing Method	Cross-Linking Mechanism	Application
Skin	[78]	Human fibroblast (HFF-1) and human keratocyte (HaCaT)	(i) Cell-suspended media for printing each cell (ii) Collagen solution for printing the supportive layer in between each cell-printed layer	Droplet printing	Thermal cross-linking of the collagen	No data
Liver	[79]	(i) Paren-chymal cell: Human liver cancer cell line (Hep G2) (ii) Non-parenchymal cells: human umbilical vein endothelial cell (HUVEC)	(i) Cell-suspended media for printing the cells (ii) Fibronectin-gelatin solution for printing the adhesive film in between each cell monolayer	Inkjet printing	Direct cell adhesion to fibronectin and gelatin	Test of hepatotoxicity of troglitazone (Rezulin)
Liver	[80]	(i) Paren-chymal cell: human-induced pluripotent stem cell-derived hepatic progenitor cells (hiPSC-HPCs) (ii) Non-parenchymal cells: HUVEC and adipose-derived stem cell (ADSC)	(i) Gelatin methacrylate (GelMa) for printing hiPSC-HPCs (ii) GelMA with glycidal methacrylate-hyaluronic acid for printing the supporting cells	Stereo-litho-graphy	UV-mediated cross-liking of the GelMA part	Test of hepatotoxicity of antibiotics, rifampicin
Liver	[81]	(i) Paren-chymal cell: Human hepatocyte (ii) Non-parenchymal cells: hepatic stellates and HUVEC	(i) Hepatocyte aggregates (ii) NovoGel for printing the non-parenchymal cells	Extrusion printing	Thermal cross-linking of the NovoGel	Test of hepatotoxicity of trovafloxacin and levofloxacin
Breast cancer	[82]	(i) Human breast cancer cell (ii) Breast stroma cells: adipocyte, mammary fibroblast, and endothelial cell	(i) Breast cancer cell aggregates (ii) Stroma cells aggregates	Extrusion printing	Self-assembly of the cells	Test of chemotherapeutic effect of tamoxifen
Cervical cancer	[83]	Human cervical cancer cell (Hela)	Gelatin-alginate-fibrinogen solution for printing the cells	Extrusion printing	(i) Thermal cross-linking of the gelatin part at 25°C (ii) Chemical cross-linking of the alginate part by CaCl_2_ solution	(i) Drug: Therapeutic effect of anticancer drug paclitaxel (ii) Assay: Cell viability

**Table 2:** Summary of 3D printed organs-on-chips with microfluidics.

Target Model	Ref	Cell Types	Bioinks	Fabrication Methods for Microfluidic Device	Applications
Liver	[84]	Hep G2 cell	Alginate solution (CaCl_2_ cross-linking)	Soft lithography for polydimethylsiloxane (PDMS) substrate and etching for glass cover slide with microchannels	Test of drug metabolism of 7-ethoxy-4-trifluoromethyl coumarin into 7-hydroxy-4-trifluoromethyl coumarin
Liver	[85]	Human liver cancer cell lines (Hep G2 and C3A)	GelMA containing pre-formed hepatic spheroids (UV cross-linking)	Casting of three PDMS chambers with microfluidic channels	Test of hepatotoxicity of acetaminophen
Liver	[87]	(i) Parenchymal cell: Hep G2 (ii) Non-parenchymal cell: HUVEC	Gelatin or collagen solution (thermal cross-linking)	One-step fabrication by extrusion printing with polycaprolactone (PCL)	No data
Nervous system	[88]	(i) Rat embryonic hippocampal neuron and sensory neuron (ii) Rat Schwann cell (S16) (iii) Porcine kidney epithelial cell (PK-15)	Cell suspension	One-step fabrication by extrusion printing with PCL, grease, and silicone	Study on pseudorabies virus infection in the nervous system
Kidney	[50]	(i) Human immortalized proximal tubule epithelial cell (RPTEC/TERT1) (ii) Support cells: human neonatal dermal fibroblast	Fibrinogen-gelatin-CaCl_2_-transglutaminase solution containing fibroblasts	One-step fabrication by extrusion printing (i) Gasket part: silicone (ii) Tubule part: pluronic F127-thrombin solution as a fugitive ink	Test of nephrotoxicity of cyclosporine A
